# Changing discourses of Chinese language maintenance in Australia: unpacking language ideologies of first-generation Chinese immigrant parents from People’s Republic of China

**DOI:** 10.3389/fpsyg.2023.1259398

**Published:** 2024-01-11

**Authors:** Yining Wang, Jia Li

**Affiliations:** ^1^Department of Linguistics, Macquarie University, Sydney, NSW, Australia; ^2^English Language Department, Guangxi Minzu University, Nanning, China; ^3^Collaborative Innovation Union for Language Governance Research, English Department, Yunnan University, Kunming, Yunnan, China

**Keywords:** heritage language, Chinese, pride, profit, Australia, language ideology

## Abstract

Parental agency of their children’s language learning is often determined by their perceptions of the significance of the language in both family and society levels. Based on a larger ethnography conducted in Sydney from 2017 to 2020, this study investigates the language ideologies of Chinese immigrant parents from the People’s Republic of China in the recent decades, regarding the maintenance of their children’s Chinese heritage language(s). Drawing on the concept of language as pride and profit shifting between communities across time and space, this study reveals that Chinese parents primarily emphasize the economic benefits associated with Chinese languages when it comes to preserving their heritage language(s). While the significance of cultural pride and identity remains evident, there is a notable shift where the concept of pride is merging with that of profit concerning the importance of Chinese heritage language. However, the commodification of Chinese and identity, privileging “national” mandarin while marginalizing “regional” others, impedes the transmission of diverse Chinese heritage languages other than Mandarin. Simultaneously, the value-laden calculation of language prioritizes the “most” prestigious English, often at the expense of “heritage” Mandarin, regardless of its acknowledged economic potential. The findings illustrate how language ideologies and practices within the Chinese diaspora are shaped by power conflicts between English and Mandarin Chinese, hierarchical distinctions between Mandarin and non-Mandarin Chinese, and subtle stratification within regional Chinese languages. The research underscores the challenges faced by minority communities in preserving their heritage languages, particularly those with limited economic capital or political influence.

## Introduction

Australia has gained a prominent reputation as a desirable destination for migration, boasting a vibrant multilingual and multicultural society. The continuous inflow of immigrants remains a defining characteristics of Australian society (Romanowski, 2021). As of 2021, approximately 7.5 million people in Australia, which accounts for 29.1% of the total population, were born overseas (Australian Bureau of Statistics, 2022). Notably, the number of Chinese-born people experienced a significant surge in the 21st century, owing to increased intakes of skilled migrants and students (Australian Bureau of Statistics, 2021). From 2011 to 2021, China consistently maintained its position as the third-largest source of immigrants to Australia (Australian Bureau of Statistics, 2017, 2021). The Chinese population proportion experienced a notable increase from 1.5% (318,969) in 2011 to 2.2% (509,555) in 2016 (Australian Bureau of Statistics, 2017). This percentage remained constant at 2.2% (549,618) in 2021 (Australian Bureau of Statistics, 2021). The recent growth stagnation may have been influenced by the impact of COVID-19-related travel restrictions in China during 2020 and 2021. The arrival of new immigrants from diverse regions of China not only enriches the dynamics of multicultural Australia in terms of ideologies, identifications, and orientations but also significantly shapes the linguistic landscape of Chinese diaspora in Australia (Wang et al., 2023).

China is a nation characterized by its rich demographic and linguistic diversity, encompassing an impressive array of approximately two thousand Chinese language varieties (Li D. C. S., 2006). The umbrella term “Chinese” consists of seven major dialects or varieties: Mandarin (originating from the north), Yue (including Cantonese), Wu (including Shanghainese), Kejia (also known as Hakka), Min (commonly referred to as Hokkien), Xiang (from the Hunan region), and Gan (from the Jiangxi region) (Taylor and Taylor, 2014). By the end of the previous century, Cantonese had been the *lingua franca* within the Chinese diaspora in Australia (Jupp, 2001). However, with the advent of the new millennium, Mandarin has steadily risen to prominence, overtaking Cantonese to become the dominant language within the community (Wang, 2020). Over the past decade (2011–2021), Mandarin Chinese has emerged as the most widely spoken language other than English in Australian households, experiencing a significant surge in speakers from 336,410 in 2011 to 685,274 in 2021 (Australian Bureau of Statistics, 2017, 2021). Although Cantonese continues to rank among the top five languages spoken at Australian homes, its number of speakers has witnessed limited growth, rising from 263,673 in 2011 to 295,281 in 2021 (Australian Bureau of Statistics, 2017, 2021). As a result, the disparity between the number of Mandarin and Cantonese speakers in Australia has become substantial. Despite a relatively minor difference in 2011, the number of Mandarin speakers now surpasses that of Cantonese speakers by a wide margin, more than doubling in comparison (Australian Bureau of Statistics, 2017, 2021).

In the context of existing studies, the classification of a heritage language can be linked to a non-societal language spoken within the home environment, readily accessible to young children, or one that maintains an ancestral connection with them (Rothman, 2009; Leeman, 2015). In alignment with this definition, the term “Chinese heritage language” in this study is employed as a broad reference for diverse Chinese language varieties (e.g., Mandarin, Cantonese, Shanghainese, Sichuanese, Hokkien, or others), whether spoken within the home domain or connected to parents’ mother tongues, even if not spoken by the children themselves. This study specifically delves into the tensions associated with preserving these different types of Chinese heritage varieties. To provide clarity, we utilize three terms: “Chinese heritage language” for general reference, “Chinese heritage language(s)” to encompass one or more varieties, and “Chinese heritage languages” when referring to multiple varieties.

The linguistic transition within the Chinese diaspora can be attributed to a range of factors, including shifts in the demographic makeup of Chinese immigrants, which no longer predominantly originate from Cantonese-speaking regions in southern China (Wang, 2020). Nevertheless, the evolving linguistic landscape of the Chinese diaspora must also take into account the language ideologies held by Chinese immigrant families (Wang, 2020) as well as the power dynamics at play within niche language markets (Curdt-Christiansen, 2018). In the ever-evolving realm of the global economy, numerous languages are gaining importance to target specific customer segments and create new forms of added value within an increasingly commodified linguistic market (Heller, 2010; Heller and Duchêne, 2012). Given China’s remarkable economic growth over the past decades, Chinese, particularly Mandarin Chinese, is progressively being recognized as a valuable asset in terms of technical skills, offering individuals enhanced prospects in education and employment (Curran, 2021). Heritage language maintenance, specifically for Chinese, finds itself at a crossroads in the twenty-first century. On one hand, the common pattern of language shift, from minority languages including Chinese to majority languages like English, continues to prevail within diasporic transnational families regarding children’s bilingual development (Wang, 2020 Piller and Gerber, 2021). On the other hand, the growing global importance of Mandarin Chinese has resulted in an increasing demand for the maintenance and acquisition of the Chinese language, as reflected in the discourse of the broader Chinese diaspora (Curdt-Christiansen, 2014; Zhang, 2020).

Immigrant parents with transnational capital often play an active role in making decisions on which language(s) their children should invest in, let go of, or even prohibit, considering the potential impact on their educational and employment prospects within and across national borders (Curdt-Christiansen and Wang, 2018; Fuentes, 2020). This study aims to investigate the ideology and agency of Chinese immigrant parents concerning their children’s language development, while considering the evolving status of Chinese languages in Australia and the wider contexts. It specifically focuses on understanding how interplay between the growing influence of Mandarin Chinese, the advantages of English proficiency, and the level of commitment to preserving non-Mandarin Chinese languages manifests within the Chinese diaspora. Thus, this research provides valuable insights into the complex and, at times, conflicting decision-making processes undertaken by Chinese immigrant parents regarding their children’s languages, identities, education, and employment. Situated in the overarching “superdiversity” trend in the broad immigrant population, this research contributes to a deeper understanding of the power dynamics that shape Chinese-English bilingualism and the local Chinese language ecology (e.g., Mandarin, and other layered Chinese varieties), within the context of Australia and beyond.

## Language as pride and profit as a theoretical framework

### Pride and profit embodied in language ideologies and practices

In order to explore the intricate implications of Chinese language usage within the context of the changing demographics of Chinese immigrants in Australia, alongside China’s increasing socioeconomic and political influence globally, we employ the theoretical framework of language as both a source of pride and a means of profit. This conceptual framework, pioneered by Duchêne and Heller (2012), reconfigures the shifting paradigm by moving away from the traditional discourse that associates language with pride and instead embraces the emerging discourse that recognizes language as a valuable tool for profit.

The ideology of seeing language as pride, usually bound up with concepts of modernity and nationalism, portrays language and culture through national and political lenses. This perspective places emphasis on aspects like heritage, rights and citizenship and associates them with the formation of nation-states (Heller and Duchêne, 2012). Within this context, elements such as cultural inheritance, linguistic preservation, and a sense of belonging are defined and casted as expressions of “pride”. These expressions serve to distinguish community members from “others” outside the community (Curdt-Christiansen and Huang, 2021). More specifically, “pride”, “as a marker of minoritized identity,” becomes associated with one’s affiliation to the cultural group, their attachment to a specific geographical place, and their relationships with family members (Tuktamyshova and Kirillova, 2023, p. 35). In a study by Blackledge and Creese’s (2012) on a Bengali community in the United Kingdom, Bengali, the national language of Bangladesh, is regarded as the symbolic representation of Bangladeshi heritage. Interviews with parents, administrators and teachers in Bengali heritage schools reveal a shared belief in the necessity for children to learn Bengali as a means of perserving knowledge of their Bangladeshi “roots” (Blackledge and Creese, 2012). This “pride”-based language orientation can also be observed in various diasporic communities and community schools worldwide (Curdt-Christiansen and Hancock, 2014; Curdt-Christiansen and Huang, 2021).

In contrast, the ideology of language as profit, often associated with new neoliberalism or late capitalism. The trope of profit regards linguistic and cultural resources as exchangeable assets with measurable value in economic terms (Heller and Duchêne, 2016). This pragmatic ideology perceives language as a product, language as a property, and language as an embodied skill (Pujolar, 2018). Embedded within this profit discourse is the commodification or marketalisation of language, prioritizing economic rationale in promoting any given language or language variety for its economic potential within neoliberal market (Leeman and Martínez, 2007).

Neoliberalism entails the marketalisation of all aspects of life, extending well beyond nation-states (Canagarajah, 2020). This ideology often involves the commodification of languages, where language becomes a commodity with varying degrees of value, creating hierarchies among languages (Sharma and Phyak, 2017). The language industry exemplifies one of the key features of late capitalism, which goes beyond national market regulations, transcending fixed identities, territorial boundaries, and the nation-state system (Heller and Duchêne, 2012). Consequently, the neoliberal commodification of language, rooted in the concept of language as a source of profit, can be viewed as a crucial phenomenon within the context of late capitalism in the age of globalization.

The global dominance of neoliberal political conditions increasingly shapes language ideologies, policies, and practices as enacted across diverse conditions and domains (Sharma and Phyak, 2017), such as in Francophone areas of Canada (Heller and Bell, 2012), in education forums in Singapore (Tupas, 2015), in media discourses of Ireland (Liu and Gao, 2020). As the commercialisation of language continues to gain prominence, a significant shift in perspective has occurred, moving away from viewing language primarily as a marker of ethnonational identity towards recognizing language as a marketable commodity in its own right (Heller, 2003, p. 474). For example, the pride associated with francophone identity remains significant, yet it has been reframed within a new discourse where proficiency in French becomes a prerequisite for employment in a job market that functions substantially in French (Heller and Bell, 2012). In many cases, efforts to promote and revitalize minority languages (e.g., Corsican) has been taken out of official and institutional contexts and are now integrated into a “poeticization of the economy” (Jaffe, 2019, p. 10).

Within the context of preserving minority languages, the interplay between language ideologies centered on pride and profit often intertwines. This dynamic relationship is notably manifested in the promotion of heritage tourism, where indigenous languages and associated semiotic elements are strategically employed as selling points to market specific destinations to tourists for both entertainment and educational purposes, all in the name of authenticity and purification (Dlaske, 2014; Jaffe, 2019; Tuktamyshova and Kirillova, 2023). Driven by the abovementioned concept of “poeticizing the economy,” the Corsican village of Pigna, for example, markets its local heritage by showcasing Corsican music performances and utilizing multilingual texts as tools to attract tourists and visitors (Jaffe, 2019, p. 11). While this commodification of such minority distinction serves economic purposes, it also integrates into a broader process of Corsican language revitalisation. The language practices associated with Corsican exemplify the discourses of both pride and profit, as similarly attested in the promotion of other minority languages, such as Tatar in Russia (Tuktamyshova and Kirillova, 2023). In the city of Kazan, the tourism industry often packages Tatar-enhanced features to offer cultural experiences to tourists and generate economic profit, which in turn creates “a stronger sense of pride, identity, and empowerment” for Tatar heritage speakers. These intertwined tropes of “pride” and “profit” are used to justify the importance of linguistic varieties, encouraging or convincing people to speak them, learn them, support them, or even pay to hear them (Heller and Duchêne, 2012).

However, the ideological tropes of language as sources of both pride and profit can come into conflict with each other. As languages are increasingly commodified as technical skills, the profit-oriented aspect appropriates the pride trope, leading to tensions and conflicts. These tensions arise when certain linguistic entities were legitimized while others being traversed (Heller and Duchêne, 2012). Take the aforementioned Bangladeshi heritage language for example. Bengali, as the language spoken by the educated elite in Bangladesh, holds significant value as a cherished heritage language within the diaspora. In contrast, Sylheti, spoken in rural areas of northeast Bangladesh, is often associated with lower social status and is perceived as indicative of a less-educated group (Blackledge and Creese, 2012). In that research, speakers of the prestigious Bengali were unwilling to allow the lower status language, such as Sylheti, to “contaminate” their linguistic resources (Blackledge and Creese, 2012). Such language categorisations highlight the dilemma confronting heritage language education in Britain. It finds itself torn between modern discourses focusing on linguistic and cultural preservation and perspectives shaped by late capitalist tendencies regarding it as a source of distinction and capital (Heller and Duchêne, 2012). The linguistic inscription of pride into profit, which privileges certain sets of linguistic resources, can perpetuate hierarchical orders between languages and reinforce class differences among speakers (Curdt-Christiansen and Huang, 2021).

### Pride or profit in the field of learning Chinese as a heritage language

When the emphasis on profit supersedes that of pride in the realm of (heritage) language education, language planning and policies tend to align with the social prestige, educational opportunities, and socioeconomic advantages associated with the language, whether at the state or family level (see Tupas, 2015; Curdt-Christiansen, 2016; Tannenbaum and Yitzhaki, 2016). In Singapore, Mandarin, as one of the four official languages, had long been celebrated for its cultural significance to the dominant Chinese population, encompassing their traditions, customs, and ancient civilisations. While Mandarin continues to serve as a language of cultural or ethnic identification in official and popular discourse, recent state policy pronouncements seem to redefine Mandarin as a valuable economic asset in addition to its heritage promotion (Tupas, 2015). In other words, besides as a medium for the transmission of culture, Mandarin serves as a pragmatic tool for achieving economic gains, a role previously reserved for English only (Tupas, 2015). The commodification of Mandarin, as represented in the bilingual policy, is part of the government’s strategic response to the growing economic and political clout of China on the global stage (Tupas, 2015). The policy reorientation indicates a potential ideological shift, viewing Mandarin as being a commodity with significant economic value, away from as being an entitlement of heritage and identity historically. In the context of family dynamics, the desire of parents to maintain the Chinese language is largely driven by external factors, particularly economic considerations (Wang, 2020). Mandarin Chinese is often viewed as a valuable asset that can lead to personal betterment and financial advantages, rather than being appreciated for its intrinsic linguistic features, literary prowess, or cultural heritage (Wang, 2020). At the grassroots level, individuals of Chinese ethnicity who come from non-Mandarin-speaking backgrounds (such as Hokkien or Cantonese) within the broad Chinese diaspora often opt not to prioritize the acquisition of their mother tongue. Instead, they make the decision to have their children learn Mandarin, aiming to gain academic recognition or improve their prospects for local and transnational employment (Zhang and Slaughter-Defoe, 2009; Curdt-Christiansen, 2016). Family language policies and decisions go beyond a mere selection of which language to learn; they unveil the power imbalance of languages and the inequalities in language usage, despite all languages being integral to the lives of transnational families (Curdt-Christiansen and Huang, 2021).

In the process of commodifying the Chinese language, Mandarin has been exclusively highlighted as the benefit reaper from China’s economic and political growth. Despite Cantonese historically being the dominant language in Chinese communities outside of China, Mandarin has now taken precedence, becoming the *lingua franca* among the broad Chinese diaspora, including in the United Kingdom (Curdt-Christiansen and Huang, 2021), Singapore (Tupas, 2015), Ireland (Liu, 2022), and Australia (Wang, 2020). Consequently, the expanding influence of Mandarin Chinese, coupled with the ideological transformation emphasizing profit orientation, is likely to significantly reshape the language ecology within the Chinese diaspora.

Research on the motivation for Chinese heritage language maintenance has identified the paradigm of economic pursuit and identity concerns as two important factors (Wang, 2020 Zhang, 2020). However, little attention has been given to the intimate interplay of power and heritage within the sphere of parental language ideologies and family language policies, encompassing the entire spectrum of langages present in immigrant families’ domain. Building upon previous research, this study delves deeper into the nuanced manifestations of power and heritage reflected in language ideologies, practices, and decisions, specifically concerning the intimate languages^1^ within individual’s lived experiences. Drawing on the theoretical framework of language as both a source of pride and profit, the study seeks to addresses the following questions:

1. What language ideologies are observed among Chinese immigrant families regarding the transmission of Chinese heritage language(s)?
2. How does the discourse surrounding speaking Chinese as a source of pride and/or profit influence the language ideologies of newly arrived Chinese immigrants?
3. What tensions, if any, are observed in maintaining Chinese as a language of profit and/or pride?

## Methodology: a critical sociolinguistic ethnography as approach

This study is based on a larger ethnography conducted with 31 Chinese families in Australia between June 2017 and May 2020 (Wang, 2020). Adopting a critical sociolinguistic ethnography (CSE) (Heller, 2008; Heller et al., 2017), the research aims to explore the multifaceted interaction of language ideologies in current diasporic settings and evolving political landscapes in both their home and the host countries. By employing CSE, this study offers an insider’s perspective on the experiences and ideologies of immigrant parents, situating them within broader social and political contexts (Motaghi-Tabari, 2016). The CSE approach provides an insider’s account of what is happening in a particular society or group and helps place immigrant parents’ lived experiences and their ideologies within wider social and political landscapes (Motaghi-Tabari, 2016). The open and context-sensitive nature of CSE allows for data collection from diverse sources and facilitates active interactions between the authors and the participants, enabling the data collection process to evolve and mature over time (Blommaert and Jie, 2010).

### Participant backgrounds

The ethnography conducted for the previous research which encompassed 31 Chinese families, involving 27 parents (23 mothers and 4 fathers) and 32 children from mainland China. This distinctive gender imbalance, which aligned with observations made by other researcher (e.g., Piller, 2002; Torsh, 2020), can be attributed to the traditional gender roles within families. This traditional framework often takes mothers as the primary caregivers and fathers as the primary breadwinner, a dynamic that was also evident in many of the families we studied. Furthermore, the willingness of mothers to participate in the study may have been influenced by my own identity as a female researcher, potentially fostering a closer connection between us.

The earlier study aimed to include at least one parent and one child from each family; however, not all parents within each family were accessible for data collection. Specifically, these 27 parents represented 24 distinct families. Among these families, certain ones featured the participation of both parents, exemplified by cases like Mother 3 and Father 3 from Family 3, Mother 18 and Father 18 from Family 28, and Mother 23 and Father 23 from Family 23. Conversely, several other families (namely, Families 22, 24, 27, 28, 29, 30, and 31) solely provided child participants. The numbering sequence assigned to parents (as shown in Table 1) was aligned with the family order. For instance, examples like Mother 3 and Father 3 were associated with Family 3. The numbering sequence was positively related to the ages of their children at the time of arrival in Australia, with families having younger arrival children assigned to the earlier numbers, while those with later arrivals were assigned to the latter numbers. Given that the central focus of the present study primarily focuses on parents, the profile table exclusively encompasses information pertaining to the participating parents.

**Table 1:** Sociodemographic profile of the Chinese parents.

Participants	Year of arrival	Educational level	Premigration occupation	Postmigration employment	Parents’ mother tongue	Language(s) with children (based on the reported preference order)
Mother 1	1992	TAFE	Pathologist	Housewife	Mandarin	English, Mandarin
Mother 2	2012	High School	Self-employed	Self-employed	Mandarin	Mandarin
Mother 3	2010	Bachelor	Nurse	Nurse	Fujiannese	Mandarin, English
Father 3	2010	Bachelor	Lawyer	Laborer	Funjiannese	Mandarin, English
Mother 4	2012	Bachelor	Manager	Self-employed	Hakka	Mandarin, English
Mother 5	2010	Bachelor	Programmer	Housewife	Cantonese	Mandarin, Cantonese, English
Mother 6	2015	Bachelor	Financier	Housewife	Shanghainese	Mandarin
Mother 7	2013	Bachelor	Accountant	Casual accountant	Mandarin	Mandarin, English
Mother 8	2007	Bachelor	Accountant	Auditor	Cantonese	Mandarin, Cantonese, English
Mother 9	2005	Bachelor	Internal auditor	Community social worker	Shanghainese	Mandarin, Shanghainese, English
Mother 10	2004	Bachelor	Purchasing officer	Settlement coordinator	Mandarin	English, Mandarin
Mother 11	2013	Master	Educational consultant	Migration advisor	Cantonese	Cantonese, English, Mandarin
Mother 12	2013	Bachelor	Manager	Housewife	Mandarin	Mandarin, English
Mother 13	2016	Master	IT engineer	Housewife	Mandarin	Mandarin
Mother 14	2000	Medical Doctor	Professor	TCM practitioner	Mandarin	Mandarin, English
Father 15	2012	Doctor	University lecturer	Childcare educator	Mandarin	Mandarin, English
Mother 16	2009	Bachelor	Salesperson	Housewife	Mandarin	Mandarin
Mother 17	2015	Master	Medical expert	Housewife	Sichuannese	Mandarin
Mother 18	2016	Bachelor	Social worker	Housewife	Shanghainese	Mandarin
Father 18	1990	Bachelor	Manager	Self-employed	Shanghainese	Mandarin
Mother 19	2014	Master	University lecturer	Housewife	Mandarin	Mandarin
Mother 20	2016	High School	Real estate agent	Cashier	Cantonese	Mandarin
Mother 21	2010	Medical doctor	Medical expert	Histologist	Mandarin	Mandarin, English
Mother 23	2015	Master	Sales manager	Pathology collector	Mandarin	Mandarin
Father 23	2015	Master	Doctor	Pathology collector	Mandarin	Mandarin
Mother 25	2003	Master	University lecturer	Business owner	Sichuannese	Mandarin, English
Mother 26	2015	Bachelor	Salesperson	Waitress	Mandarin	Mandarin

These participating parents, who can be considered first-generation Chinese immigrant parents, arrived in Australia in the recent decades, spanning from the 1990s to 2016. The term “first-generation Chinese immigrant parents” in the study refers to individuals who were born in China and later moved to another country like Australia, making them the initial generation of their families to settle in Australia. Specifically, the majority of these parents (25 out of 27) arrived in Australia between 2000 and 2016, while only two had immigrated in the 1990s (see Table 1). Although their length of stay in Australia varied considerably, ranging from 1 to 25 years, most of them (19 out of 27) had been residing in Australia for over 4 years at the time of the initial interviews in 2017, with an average duration of 7 years (see Table 1). It is important to acknowledge that the diverse migration periods and durations of residence in Australia are likely to influence their language beliefs and practices. For instance, those who migrated in the 1990s appeared to hold distinct attitudes regarding the importance of maintaining Mandarin Chinese when compared to those who arrived in the most recent decade (see the section of findings). The research endeavors to mitigate the potential impact of the factors related to parental migration years and duration. This effort is apparent in the shared migration experiences of the majority of participants, who arrived within the past two decades and resided in Australia for several years before data collection. Additionally, the selection of these families is characterized by children who have similar migration histories, specifically those who immigrated between the ages of 3–10. This selection criterion was adopted for the sake of convenience in investigating parents’ language ideologies and practices.

The majority of these parents were well-educated and held professional positions before they immigrated. Among the 27 parents, 13 held a Bachelor’s degree, 8 a Master’s degree, and 3 a doctorate, most of which were obtained in China. Prior to migration, most of these parents worked in professional roles in academia, government, finance, IT, or medicine. However, following migration, many experienced a decline in occupational status and encountered significant challenges in securing employment in their respective fields. At the time of the interviews, only two parents had re-established themselves in positions within their previous professions, while eight parents became homemakers, and others had taken on lesser roles in childcare, social work, construction or were self-employed. The educational background of these parents, coupled with the economic and career disparities before and after migration, can be considered a contributing factor that led to their aspiration for their children’s socio-economic advancement through education, including language learning (refer to the analysis sections).

### Data collection methods

The research data comprised open-ended semi-structured interviews conducted with parents, along with fieldnotes derived from informal conversations and observations, and background questionnaires. Mandarin served as the default language for interviews with all parents, except for two individuals who opted to switch to English a brief moment into the interview. These interviews were audio-recorded, transcribed verbatim, and meticulously proofread by the two researchers. If the selected transcripts were in Chinese, they were translated into English for analysis. All direct quotes employed in the research are either translations or transcripts in English.

The interview questions encompassed a range of topics, including children’s Chinese language learning experiences both before and after migration, parents’ expectations regarding the heritage language and children’s education, and family language policies implemented by Chinese immigrant families (see details in Interview Guide). As an extension of the interviews, the inclusion of “ethnographic nature” voices (Lee, 2014) from private communications aimed to capture participants’ diverse and evolving thoughts, ideologies, and circumstances. These informal conversations were collected through private meetings and via postings and exchanges on WeChat (a multifaceted platform that combines instant messaging with social networking features). Furthermore, the background questionnaire, formulated in English, comprised inquiries about demographic information, linguistic backgrounds, and language use within participant families’ households (see details in Family Questionaire). The questionnaire was distributed to participants for completion at the outset of the interview session. The design and sequencing of the questionnaire, preceding the interview, helped Author 1 (the data collector) to have a rough understanding of the parents’ linguistic situation. Consequently, this understanding enabled her to adjust interview questions based on individual circumstances. The background knowledge has aided in establishing profiles for the participating families.

In the collected data, the parents predominantly used the term “the Chinese language” to refer to Mandarin, unless specifically stated otherwise. The term “Mandarin” itself was seldom utilized, except when distinguishing it from other varieties of Chinese. This linguistic habit among the first-generation Chinese immigrant parents may stem from Mandarin’s esteemed status as the *lingua franca* in mainland China. Therefore, unless explicitly specified, the phrase “the Chinese language” mentioned in the excerpts within this study should be understood as referring to Mandarin.

Negotiating our roles, whether as friends, researchers, or participants, was essential in a context where language was a central topic of discussion (Morgan, 2017). In the research, the fieldwork was conducted by Author 1, who was herself a migrant parent at the time of data collection. Author 1’s insider position played a significant role in fostering in-depth discussions with the participating parents regarding topics such as language attitudes, heritage language maintenance, and bilingual parenting. Most of the interviewed participants perceived Author 1 both as a fellow parent and as a researcher. They exhibited a strong willingness to share their experiences of bilingual parenting and to collaboratively explore effective strategies for providing their children with bilingual support. Simultaneously, once they became aware of Author 1’s situation as a new migrant, they also viewed her as a novice parent within a migration context. Consequently, they generously imparted their knowledge about the Australian educational system to her. In fact, during the subsequent analysis, this insider identity, which had allowed Author 1 to align with parental linguistic stances, enabled a deeper understanding of the emotional aspects underpinning their language behaviors. This included their emotional struggles and policy inconsistencies when attempting to maintain their children’s proficiency in Mandarin Chinese while simultaneously supporting their academic excellence in school (see the findings section).

### Data analysis

Building on previous ethnographies as models (e.g., Curdt-Christiansen and Wang, 2018; Fuentes, 2020), we employed a combined approach, utilizing both deductive and inductive methods to analyse the data. Initially, we conducted a deductive segmentation of transcripts and fieldnotes based on predetermined themes that emerged as salient or were identified as relevant during the data collection process. These predetermined components, specifically “Chinese as profit,” “Chinese as pride,” “family language policies,” “occurrence of tensions,” and “families’ linguistic background,” were deemed most pertinent to our research inquiries. By categorizing the data into these segments, we established the groundwork for our analysis. Subsequently, through an inductive process, we identified and categorized emerging themes within each segment. After reading the segmented data under the pre-determined headings, we carefully examined and coded significant themes while noting their similarities and differences. Through constant comparison and contrast of the data, we merged similar themes and grouped them into primary thematic topics. For instance, within the “Chinese as profit” segment, we coded the data into themes such as “learning Chinese due to China’s rise,” “learning Chinese for job opportunities,” “learning Chinese for return migration,” and so on. Similarly, within the “Chinese as pride” segment, we identified themes such as “Chinese as identity,” “Chinese as cultural legacy,” “Chinese as family bond” and others. The “occurrence of tensions” segment yielded themes like “language policy conflicts between English and Chinese,” “language policy conflicts between Mandarin and non-Mandarin,” “language policy conflicts within non-Mandarin Chinese,” and others.

In the following section, we will elaborate on the identified themes, conceptualizing them as four designated components: Chinese as a maker of employability, Chinese as a marker of identity, Chinese as a tie of family relations, and tensions in maintaining Chinese language(s).

## Findings: language ideologies of the newly arrived Chinese immigrant parents

The interviewed parents expressed a strong desire for their children’s acquisition of the Chinese language. With the exception of Mother 10 and Mother 25, all parents had established explicit family language rules to enforce their children’s learning of Chinese, particularly Mandarin Chinese, including Chinese literacy skills. Mother 10 and Mother 25, however, admitted that they did not compel their children to continue Chinese language practice when their children expressed a dislike for the language.

Parents themselves demonstrated a proficient command of Mandarin Chinese, as it had been the language of their entire education in Mainland China. During the interviews, 11 out of the 27 parents (41%) openly revealed that they primarily spoke non-Mandarin mother tongues with their spouses and/or extended families. These non-Mandarin varieties included Cantonese, Fujianese, Sichuanese, Shanghainese, and Hakka (refer to Table 1). Despite this, all parents unanimously affirmed that Mandarin was the heritage language they actively sought to maintain within their homes. Among the 11 parents who spoke a non-Mandarin mother tongue, only four admitted to considering their own mother tongues as valuable assets for transmission. They intentionally spoke both their mother tongue and Mandarin to their children. The non-Mandarin Chinese dialects purposefully maintained at home were Shanghainese (spoken by Mother 9) and Cantonese (spoken by Mother 5, Mother 8, and Mother 11). The remaining seven parents, speaking Fujianese, Sichuanese, and Hakka, acknowledged that they had never contemplated transmitting their dialects to their children.

The parents consistently cited economic advantages, cultural identity, and family communication as the most prevalent reasons for underscoring the importance of maintaining the Chinese heritage language, as illustrated in the following (also refer to Table 2).

**Table 2:** Reasons for maintaining Chinese.

Reasons for Chinese heritage language maintenance	Total (*n* = 27)	Percentage
To achieve economic gains	18	67
To preserve Chinese identity	15	56
To maintain family relations	7	26

### Chinese as a maker of employability and mobility

Parents are highly motivated to invest in their children’s learning of the Chinese language due to its potential to enhance their employment opportunities and upward mobility. Specifically, 18 parents (67% of those interviewed) linked their aspirations for preserving Chinese language skills (more precisely, Mandarin Chinese) to the economic, occupational, and educational advantages that come with proficiency in the language.

The remarkable economic growth of China serves as a pivotal catalyst, fostering parents’ positive attitudes towards the Chinese language and reinforcing their belief in the economic returns associated with learning it. For instance, Shanghainese Mother 9 was one of the few parents who maintained their children’s non-Mandarin dialect following migration (see details in Excerpt 7). Nevertheless, the focal point of her family’s language policy remained firmly on ensuring her daughter’s mastery of Mandarin. She had diligently supported this endeavor for a decade, spanning various educational institutions such as Chinese community schools, private tutoring, and back-to-China studies. She exclusively linked her maintenance commitment to China’s socio-economic prospects on the global stage.

(1) China is the second largest economy in the world. It has business with many countries. As for my daughter, she needs to carry two major tasks on her shoulders—taking care of her English studies, and also developing her Chinese language proficiency. I said to her, “if you develop your future in China, what you can use to compete is your English; but if you stay in Australia, what you must have to compete is your Chinese.” That’s why neither her Chinese nor her English can be allowed to drag behind. (Interview) [Adapted from Wang (2023)]

The sentiments expressed by parents shed light on two significant aspects that underline the influence of power structures on language preservation. Firstly, it demonstrates how the political and economic status of a home country, such as China, empowers its social agents, specifically Chinese immigrant parents in migration contexts like Australia, to uphold their heritage languages and cultures. The above-mentioned excerpt emphasizes the socio-political and economic prominence of China on the global stage, which strengthens parents’ convictions regarding the economic potential of Mandarin Chinese and further reinforces their desire for their children to attain proficiency in Chinese. Secondly, by positioning Chinese on an equal footing with English, the universally recognized *lingua franca*, the prestigious status of Mandarin Chinese as an emerging world language appears to be well-attested and embraced within the Chinese diaspora. Throughout the gathered data, parents consistently demonstrated a discernible sense of pride in the increasing prominence of their heritage language, coupled with a sense of urgency to tap into the potential economic benefits offered by the growing Chinese market (as also evident in the following excerpts).

In light of the predominant emphasis on the economic benefits linked to learning Chinese, proficiency in the language is frequently regarded as a valuable asset for job opportunities, career advancement, socio-economic progress, and cross-border navigation. Father 18, another Shanghainese parent, communicated in Chinese with his wife and extended family, reserving Mandarin exclusively for conversations with his daughter. For him, the pragmatic utility of Mandarin represents a dedicated commitment to fostering his daughter’s Chinese language proficiency:

(2) Having Chinese means more job opportunities. China’s economy is growing so fast, and how can we predict whether she’d better develop her career in China after she grows up. So, Chinese is a must […] Even if she stays in Australia, she still needs Chinese. Chinese language skill will be an advantage in the future. See, if you speak beautiful English and have a good command of Chinese, you are obviously advantaged and you get the best of both worlds. (Interview)

From a parental perspective, though English continues to be a prerequisite for accessing many specialized markets, it is no longer sufficient to provide a competitive edge in the globalized job market. Mandarin Chinese, on the other hand, is seen as a valuable technical skill that carries the shared responsibility of enhancing career opportunities through transnational mobility (Curran, 2021). This is exemplified by the idea of relocating from Australia to China, as mentioned by Father 18. It is worth noting that transnational mobility, desired by many parents including Father 18, predominantly occurs between English-speaking and Chinese-speaking contexts. This can be attributed to the consistent identification of English and Mandarin Chinese in the data as the two most profitable languages, granting access to occupational and economic resources across various global and diasporic spaces. The prestige ascribed to Mandarin can also elucidate why Father 18 and Mother 18 opted to disregard their native Shanghainese dialect, choosing instead to communicate exclusively in Mandarin with their daughter.

Perceiving China as an emerging economic powerhouse, parents no longer view Australia as the permanent destination, but rather as a steppingstone. Return migration to China is increasingly considered as a viable option when contemplating their children’s future trajectories. Consequently, mastering the Chinese language has become an essential skill for keeping the possibility of returning to China and pursuing a prosperous career open. However, the hindered progress, or even loss, of children’s Chinese language proficiency, poses a significant threat to parents’ expectations of career advancement in both their current location and, particularly, within the context of China. This implies that the desired upward social mobility may result in an undesired outcome: the fear of “being stranded in Australia”, as articulated by Mother 7, who communicated in Mandarin, the sole Chinese variety used with all her family:

(3) For me, Australia is not the only option. China continues to develop, and there are increasing opportunities there. But if Daughter 7’s Chinese keeps deteriorating, we can go nowhere but stay in Australia. (Fieldnote) [Adapted from Wang (2023)]

These middle-class immigrant families, equipped with transnational resources on their own, aspire to climb the social ladder through transnational mobility, ideally spanning between English-dominant Western countries and Chinese-operating Eastern countries. The realization of transnational mobility is envisioned with the aid of the two pivotal languages: English, serving as a gateway to education and employment (Curran, 2021), and Mandarin Chinese, representing their instrumental heritage that is gaining prominence.

As outlined in the methodology section, it becomes apparent that differences in parents’ migration experiences, influenced by the era of their migration, have a noticeable impact on their attitudes regarding the preservation of Chinese heritage language, particularly Mandarin Chinese. In my research, parents who immigrated in the 1990s and early 2000s, such as Mother 10 and Mother 25, appeared to be less inclined to prioritize the maintenance of Chinese heritage language. Conversely, all the parents who migrated at a later stage demonstrated a strong commitment to maintaining Mandarin Chinese, often linking their language preservation efforts to the increasing value of Mandarin in the job market. This also underscores the significant role of economic considerations in shaping immigrant families’ dedication to preserving their heritage languages.

As reflected above, the growing significance of the Chinese language and China’s socio-economic influence has led to an increasing commodification of Mandarin as a heritage language. This shift in focus places greater emphasis on the economic benefits it brings in niche markets, while diminishing attention towards the concepts of heritage and identity. Mother 9 expressed this evolving perspective when she stated, “I feel Chinese is very useful and will even be super-useful in the future. This [Chinese maintenance] is more than a heritage issue” (Interview). This statement highlights the parents’ transition from an “identity-based” perspective to a “more instrumental” one (Liu, 2022, p. 15). For these parents, the “heritage” Mandarin, “en-route to becoming the world’s other *lingua franca*” (Seng and Lai, 2010, p. 25), has emerged as an indispensable “commodity” that is expected to hitch the second generation to the economic growth of their parental homeland.

### Chinese as a marker of ethnicity and identity

Parents recognized the significance of Chinese ethnicity and cultural heritage in the maintenance of Chinese heritage language, although the percentage (56%) of those emphasizing ethnic and cultural pride is slightly lower compared to those pursuing economic gains. Nevertheless, the evolving discourse of pride aligns the Chinese language with ethnicity and identity, assigning particular significance to Mandarin over other Chinese varieties. This emphasis is evident in the perceived role of Mandarin in ethnic embodiment, resistance against racism, and the transmission of cultural heritage.

The Chinese parents’ perception of language as identity appears to be deeply ingrained. They associate their children’s Chinese identity primarily with their physical racial attributes. According to their perspective, learning Chinese becomes crucial in triggering their children’s recognition of their Chinese heritage, with speaking the language seen as a symbolic expression of their racial embodiment. Mother 21, Mother 21, who maintained Mandarin as the primary spoken language with her daughter, underscored the significance of learning Chinese in terms of augmenting ethnic visibility:

(4) Learning Chinese is a must, I think, for our Chineseness. This is the root, our identity. For example, if you say you are European, you are still defined as Asian whenever and wherever you are present, because your black hair and yellow skin are fixed and unchangeable, right? (Interview)

In the aforementioned excerpt, the concept of “Chineseness” or Chinese identity is distinguished from physical racial characteristics such as “black hair” and “yellow skin”. Speaking Chinese is regarded as the legitimate means by which the visible embodiment is linked to symbolic identity. This perspective on the language-identity connection aligns with Francis et al.’s (2014) observation that the racially marked body, identified as Chinese, is expected to embody Chineseness as defined in the imagined community, particularly through the reproduction and maintenance of the Chinese language. Furthermore, the parents are acutely aware of the potential racist discrimination that their children might encounter in a migratory context, drawing from their own lived experiences and deep connection to their cultural identity. For Mother 4, who hailed from a Hakka-speaking community, Mandarin served as the exclusive Chinese she shared with her daughter, and she was also devoted to nurturing her daughter’s proficiency in Chinese literacy. When inquired about the motivation, she passionately conveyed that the foundation for resilience against racism lies in fostering “ethnic awareness and ethnic pride in your own culture,” and, most importantly “embracing one’s true self” within a diverse and multicultural society. Likewise, Shanghainese Father 18 exclusively conversed in Mandarin with her daughter, advocating for the significance of learning Chinese and perpetuating Chinese culture as a response to situations involving racism.

(5) Even though you might be brought up here, there definitely exists differences between you and them, no matter how well you speak English. It is impossible for you to be regarded as one of them. That’s why these Chinese-born children need their own language and culture. (Interview)

The identity of Chinese migrant children is often seen as contrasting with that of local Australians, as highlighted by Father 18 who consistently referred to the former as “you” and the latter as “them”. According to him, despite the Chinese children being fluent in English like their local counterparts, they are not accepted as part of the in-group. This distinction in identity, primarily rooted in racial characteristics, becomes a significant source of exclusion, racism, and discrimination. Being visibly Chinese creates a barrier to inclusion and exposes them to othering, which is why parents believe that learning Chinese, particularly Mandarin Chinese, serves as a powerful tool for withstanding potential racism. In this context, the specific Mandarin Chinese and the broader Chinese culture act as a “protective shield” for children to rely on when faced with racial identity issues (Jacobson, 2008, p. 75). This motivation to learn Chinese as a defense against exclusion and racism aligns with the parental drive to have their children study Korean in Shin’s (2013) research, where mothers in the study aimed to cultivate a positive racial identity in their children, anticipating that it would prepare them for encounters with racism.

Besides fostering a positive racial identity, the discourse surrounding the acquisition of Mandarin is also influenced by Chinese parents’ deep admiration and pride for the aesthetic, cultural and literary prowess of the Chinese language. In the data, Chinese is often celebrated as a “beautiful language” with a rich “5,000-year-long history,” and its complex writing system is frequently regarded as one of the most challenging languages to master. These aspects are often cited as evidence of the invaluable cultural heritage of the Chinese language, making it deserving of preservation and inheritance. It should be noted that parents habitually associated these multifaceted literary values of Chinese with their commitment to Mandarin. However, the perceived loss of its linguistic and cultural capital is seen as a hindrance to children’s complete development as legitimate or competitive Chinese Australians. Mother 1 expressed her disappointment, stating, “It’s a pity that my daughter has rejected to learn Chinese and she has limited knowledge of Chinese culture. If she could combine Chinese culture with Western culture, she would be like a tiger with wings” (Interview).

In fact, the representation of identity itself appears to be increasingly commodified, manifesting in the profit-driven pursuit of belonging evident in parents’ aspirations for return migration (see details in the previous section). This trend of commodification extends to the selection of Mandarin, rather than their native tongues, as the standardized symbol of heritage, identity, ethnicity, and belonging. Parents’ (e.g., Mother 7 and Father 18) envision their return to China, which is heavily contingent upon the preservation of Mandarin Chinese, as a reflection of their attachment to an idealized “homeland”. This heightened sense of belonging finds support in China’s remarkable economic growth, making Mandarin the most beneficial language to reap rewards. Apart from language-specific factors, the socio-economic power of one’s home country can instill in individuals residing in the host society a sense of “long-distance nationalism” or affiliation, thereby reinforcing a deep-rooted “pride in the Chinese national identity” (Curdt-Christiansen and Huang, 2021, p. 59). Within this context, the interplay between pride and profit regarding the preservation of the Chinese language for the sake of ethnicity and identity is intricately woven and mutually constituted. This dynamic suggests a tendency for a transition from pride to profit, particularly if the allure of materialistic gains within their home country continues to persist.

### Chinese as a tie of family relations

The importance of learning Chinese for the purpose of family cohesion was acknowledged by only seven of the parents interviewed (26%). The limited emphasis on Chinese in family communication can be attributed to the parents’ own proficiency in English, which allows them to comprehend their children’s English at a communicative level. Out of the 27 parents, 24 (89%) held a bachelor’s degree or higher, and on average, they had been living in Australia for 7 years. Additionally, only four parents (15%) required assistance when completing the English questionnaire at the outset of interview. These factors (e.g., educational background, migration experiences, and displayed English ability) suggest that these parents likely possess functional or even advanced English skills within their work and living environments. In fact, apart from Chinese, parents identified English as a commonly used language in family communication, particularly when their children responded to them. Nevertheless, parents still regarded Chinese as the language that conveys intimate emotions, providing a sense of familiarity and comfort. They believe that their children’s proficiency and knowledge of Chinese are crucial for fostering deep parent–child communication. As Mother 21, a typical Mandarin-speaking parent, elucidated:

(6) Compared with most Chinese migrants, our English is not bad, but the English spoken by us – first-generation immigrants, is not at all comparable with them – the second generation […] There are always things we cannot express unless resorting to our mother tongue. That’s why I think my daughter needs to learn our language. The more proficient she is in Chinese, the more freely she could express herself with it. So, we would have more freedom in conducting conversations.

Parents like Mother 21 perceive the linguistic divide between parents and children as a threat to the freedom of communication within the family. They believe that closing the language proficiency gap requires the second generation to freely express themselves in Chinese. In cases where Chinese has not been successfully maintained, conversations with parents or other family members may remain on a superficial level, potentially affecting family harmony. These linguistic disparities often result in reduced shared beliefs, values, and behaviors, becoming a noticeable source of grief and sorrow for parents. When Mother 1 expressed her sadness over the lack of meaningful conversations with her daughter, she exclaimed, “She is a foreigner to me.” Similarly, Mother 10, despite fluently conversing with her daughter in English, expressed regret that her daughter’s language loss severed the communication channel with her grandparents.

It is worth noting that during the interviews discussing the significance and practice of maintaining Chinese heritage language, Mandarin Chinese serves as the default reference language, even though many parents (11) originate from non-Mandarin speaking backgrounds (see Table 1). This implies that Mandarin, as opposed to any other Chinese dialect, is regarded as the legitimate language not only for enhancing career prospects and representing Chinese identity but also for preserving family bonds. Furthermore, it is important to clarify that among the 20 parents who do not explicitly mention the Chinese language as a factor in family relationships, their relevant language ideology does not seem notably tied to their own individual linguistic backgrounds. This means that they could be Mandarin speakers, Cantonese speakers, or speakers of any other Chinese dialects.

In summary, within parental discussions, the importance of Chinese heritage language is seen as a convergence of three key aspects: its potential for economic gain, its role in ethnic identity formation, and its ability to foster family unity. However, this viewpoint also reveals a shift towards instrumental orientations, wherein Mandarin Chinese is increasingly viewed as a valuable global commodity linked to economic growth and the market value of China. The commodification of the Chinese language is, in fact, attributed to the power relations of various Chinese languages, as they acquire symbolic capital “associated with politicized identities unified through homogenization and standardization of language and culture” (Tuktamyshova and Kirillova, 2023, p. 35). Unfortunately, the process of language standardization and commodification poses significant obstacles to the preservation of diverse Chinese heritage languages and the “legitimate” Chinese heritage language.

### Tensions in maintaining Chinese “heritage” languages and the Chinese “heritage” language

As previously mentioned, the Chinese language is incredibly diverse, consisting of thousands of varieties that are primarily classified into seven subgroups. It is important to note that most of these languages are mutually unintelligible, implying that speakers of one subgroup may not understand those of another (Curdt-Christiansen and Gao, 2020). However, as previously mentioned, all parents, regardless of their individual Chinese dialects, can use Mandarin as an effective vehicle of communication in the Chinese diaspora. Regarding the maintenance of heritage languages, the hybridity nature of Chinese raises questions about “which specific languages are being maintained by Chinese families” or “which languages are considered their true heritage languages.” As mentioned earlier, the majority of the parents aspire for their children to acquire a proficient command of Chinese, be it to enhance their job prospects or to preserve their culture and identity. It is worth noting, as mentioned earlier, that when referring to “the Chinese language,” all participants implicitly refer to “Mandarin Chinese” rather than other varieties. During our fieldwork, we identified a clear consensus among many mainland Chinese perspectives that Mandarin is regarded as a distinct language, whereas other Chinese language varieties are often viewed as regional dialects (see also Wiley et al., 2008). Although these dialects may hold economic or symbolic value, they are not regarded on the same level as Mandarin. Hence, it can be inferred that, for these parents, maintaining Chinese heritage language naturally equates to the preservation of Mandarin Chinese, regardless of their actual language backgrounds. This habitual use of terminology unveils the hierarchical distinction between Mandarin, as the prestigious national language, and other varieties, which are typically indexed to locality and lower status, as revealed in the following.

As a heritage language is often termed as an “ancestral language”, “minority language” or “community language” (Leeman, 2015), these non-Mandarin Chinese varieties spoken by the 11 parents in the research can be considered as their children’s heritage languages. However, the research data indicates earlier that only four parents regularly speak their heritage Cantonese or Shanghainese to their children, while none of the parents speak their heritage Sichuanese, Fujiannese, or Hakka to their children on a regular basis or intend to maintain these heritage mother tongues (refer to the above sections). Parents’ lax attitudes to their own spoken languages, in contrast to their dedication to Mandarin, further accentuate the distinct power disparity between the national language and regional dialects. Parents’ subtle indications and undertones regarding regional dialects unveil an implicit power hierarchy, wherein specific “regional varieties,” such as Cantonese and Shanghainese, are accorded a higher status compared to others, as indicated by Shanghainese Mother 9:

(7) We Shanghai people, more or less, have a sense of pride in being Shanghainese. So, I still want to my daughter to keep our language. But most of other Shanghai families in my circle have given up speaking Shanghainese with their children because they think Shanghainese is not that useful and Mandarin is the most important. (Interview) [Adapted from Wang (2023)]

**Figure 1:**
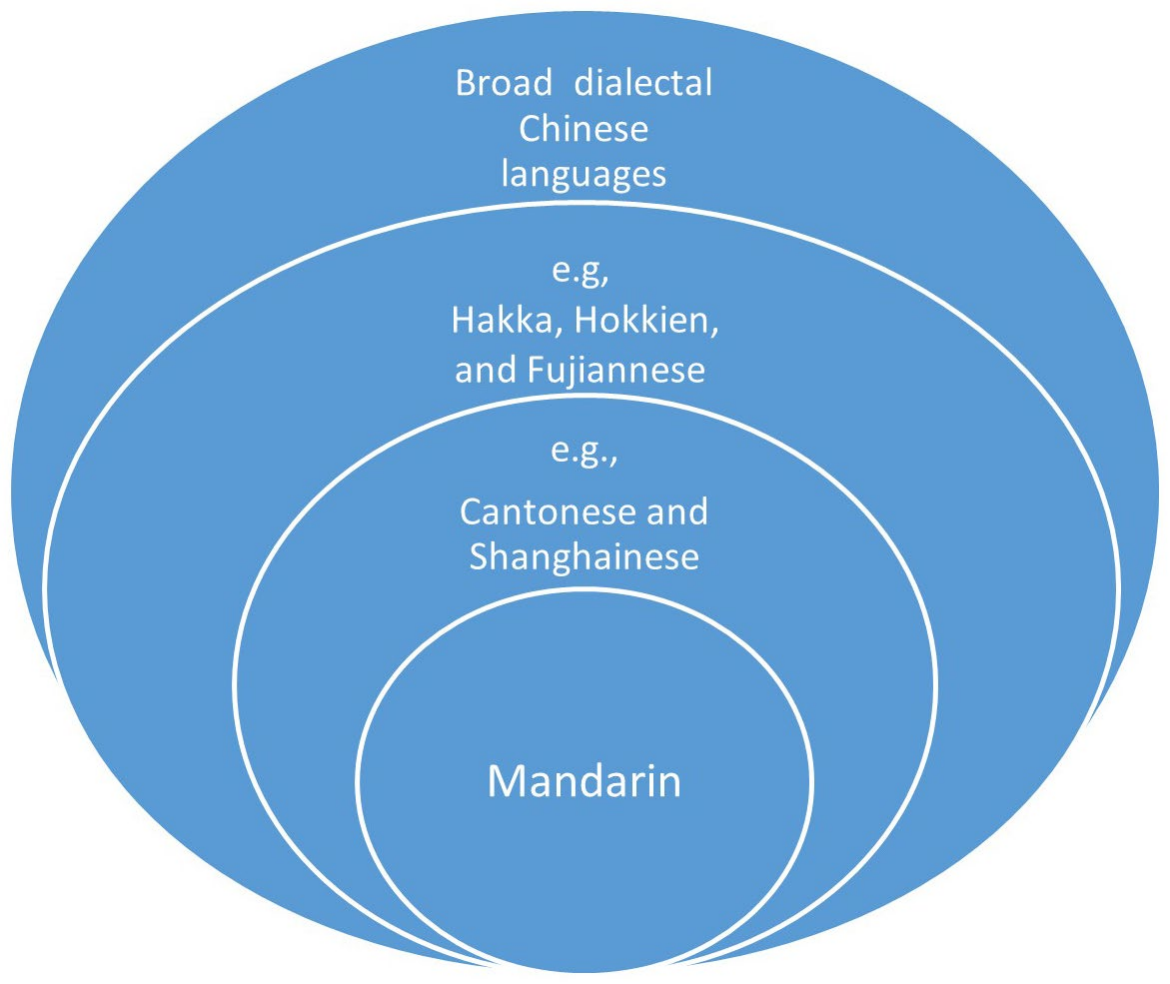
Hierarchies of Chinese languages.

Mother 9’s affection for the Shanghainese language is rooted in her pride as a Shanghainese person. During our conversations with other members of the Shanghainese community, we have observed that this regional identity, centered around Shanghai, is sometimes presented as superior to other regional identities within China. This sense of regional superiority is often associated with the perceived modernity of Shanghai, a city of iconic status in China. Similarly, parents like Mother 8 and Mother 11 have expressed their willingness to preserve both Mandarin and Cantonese as their cultural heritage. Cantonese, historically serving as the *lingua franca* in diasporic communities (Liu and Gao, 2020; Curdt-Christiansen and Huang, 2021), is also viewed by these parents as “a useful language” in the Australian job market, making it worth passing down to the next generation. Nevertheless, the prominence of both the iconicity of Shanghainese and the functionality of Cantonese are subject to the overarching prestige of Mandarin. Mandarin, being the official language the Chinese government and the medium of instruction of schools across Mainland China, takes precedence over other Chinese dialects or varieties. This prioritization aligns with the language preferences of other Chinese parents investigated both in the context of China (Curdt-Christiansen and Wang, 2018) and in immigration settings (Shen and Jiang, 2023). In addition to the hierarchical distinction between Mandarin and other Chinese varieties, our study reveals that parental (dis)favor for certain languages further accentuates nuanced and implicit stratification among different Chinese dialects (see Figure 1). Mandarin, positioned at the pinnacle of all Chinese languages, is followed by specific sets of varieties, such as Cantonese and Shanghainese in our research, owing to their economic significance, symbolic value, or functional utility. In an increasingly neoliberal market, the social status of Chinese languages is manifested through differentiated attitudes and investments made by their speakers: Mandarin receives substantial investment, while specific linguistic resources (e.g., Cantonese and Shanghainese) are deemed worthy of preservation within the family domain, while other linguistic resources, considered useless, can be foregone. This privileging of national Mandarin, at the expense of broader regional languages, poses a threat to the diversity of linguistic resources and cultural identities that have characterized Chinese diasporic communities (see also Liu, 2022).

The strong grassroots desire for Mandarin proficiency as a heritage language may lead to an optimistic assumption regarding the successful maintenance of Mandarin skills. However, our research reveals that parents often struggle to maintain consistent commitment to their children’s Mandarin learning, resulting in their Mandarin proficiency becoming stranded or hindered as their children progress to higher grades. The difficulty of maintaining desired Mandarin proficiency is deeply rooted in the power relations between Mandarin—the “legitimate” heritage language with growing capital and English—the established world lingual Franca. Our data consistently shows that, despite parents placing great value on Mandarin Chinese, they frequently interrupt their children’s Chinese learning activities to prioritize more immediate high-stakes assessments—normally English-related—such as Opportunity Class Test^2^ and Selective High School Test^3^ and HSC examination.^4^ Mother 16’s account exemplifies this paradoxical pattern, as she constantly felt compelled to halt her son’s Chinese lessons in the community school in order to ensure his academic competence during critical stages:

(8) We suspended his Chinese classes to make way for tutoring classes, in Year Four targeting at OC [the Opportunity Class test], and then in Year Five at Selective School. […] After the Selective test, he resisted going back to the Chinese community school. […] Now he seems to have interest in learning Chinese, but we need to prepare him for HSC examination, so the focus is on English and math tutoring. And Chinese needs to be missed again in our busy agenda! (Interview) [Adapted from Wang (2023)]

Becoming academically excellent in mainstream schools is the wish that these immigrant parents hold in common. When seeing proficiency in Chinese cannot be credited in school system, parents often compromise the heritage Chinese, though it carries both emotional demands and material pursuit. They choose to prioritize English, which is considered the most “profitable” language, along with subjects that are deemed more “useful” and are typically taught in English. The decision to maximize the profit by prioritizing English over Chinese appears to be an education-wise strategy, as emphasized by Mother 3, who stated that “we should prioritize what is most important.” However, parents consistently grapple with internal conflicts when it comes to relinquishing the advantages or profits that come with achieving proficiency as heritage users. For instance, the compromise of Chinese proficiency often evokes a profound sense of loss and regret, as expressed by Mother 16. Following the suspension of Chinese classes, she mourned the decline in her child’s Chinese skills, stating, “Now, his command of Chinese is deteriorating rapidly.” This feeling of loss or anxiety can be even more pronounced for parents like Mother 7, who had envisioned upward mobility through a potential return migration (as discussed earlier). In this context, the profit-oriented language policy represents both a gain and a loss in terms of “profit”. The “gain” lies in the anticipated enhancement of opportunity to excel in high-stakes tests, which significantly impact children’s immediate educational prospects. However, the “loss” primarily stems from the diminished potential for future career advancement in the rapidly growing Chinese market.

The intricate array of emotions related to heritage language exemplifies that the decision about “to learn or not to learn” is anything but straightforward for many immigrant families. It is a decision fraught with tensions as immigrants find themselves compelled to “conform to institutional structures that promote linguistic hierarchy” (Curdt-Christiansen and Huang, 2021, p. 61). Our observations reveal a profound clash between various tiers of societal and heritage language benefits, vying for ideological supremacy (Curdt-Christiansen and Huang, 2021, p. 64) within the arena of parental discourse. This conflict permeates into how profit-oriented ideologies assert their dominance, shaping the language policies and practices within families. However, the decision to prioritize one language over another goes beyond mere instrumental pursuits of parents; it delves into the deeply ingrained linguistic inequality prevalent in educational discourses and the enduring challenges faced in preserving minority languages.

As depicted in this section, FLPs reveal three distinct hierarchical orders: a global hierarchy between English and Mandarin, a national hierarchy between Mandarin and other Chinese languages, and a regional hierarchy within different non-Mandarin varieties. The profit-driven ideology compels families to prioritize languages placed at the apex of these multiple hierarchies, and the choices stemming from the three hierarchies often intersect. Mandarin holds a position of preference over any other Chinese variety or dialect, whether its symbolic Shanghainese or functional Cantonese. However, when English enters the equation, Mandarin is then displaced. This fluid positioning of linguistic varieties is also reflected in Wan and Cowie’s (2021) report on Taiwanese immigrants’ identification with and deployment of various world Englishes and global Mandarin in the context of Singapore. From the perspective of these Taiwanese immigrants, Singaporean colloquial English, perceived as a poor imitation of English, is considered hybrid, chaotic, and impure. In contrast, American English, seen as the model of all English varieties, is presented as a choice made on a national level by the Taiwanese. Additionally, Singapore Mandarin is viewed as occupying a lower status, while both Taiwan Mandarin and Beijing Mandarin are seen as standards of authenticity.

The language ideologies and decisions of the Chinese immigrants reveals how the discourse of “pride” associated with lower-ranked regional languages (e.g., Shanghainese and Cantonese) yields to the discourse of “profit” derived from higher-ranked standard languages (e.g., Mandarin). It also reveals how linguistic resources with lesser global capital (e.g., Chinese) are compelled to yield to those with higher global prestige (e.g., English). This profit-driven language ideology, rooted in the deeply ingrained yet evolving power dynamics of late capitalist conditions, significantly impacts the linguistic practices of Chinese immigrants in migration contexts. It shapes their language investment in Chinese language learning, choices between different Chinese linguistic resources, and the timing of language decisions regarding the prioritization of English over Chinese.

## Conclusion and discussion

Our research highlights two significant discourses that shape the ideological perspectives of Chinese immigrant parents in Australia: language as profit and language as pride. It reveals a shift in discourse from identity-based pride to a focus on resource-oriented profit when it comes to the motivation of Chinese language maintenance. Furthermore, the commodification of the Chinese language, which places excessive emphasis on national Mandarin while marginalizing other regional languages, impedes the transmission of diverse Chinese heritage languages other than Mandarin. Equally significant, power conflicts between English and Chinese serve as a major barrier to the preservation of Mandarin, a language that enjoys widespread recognition as the legitimate Chinese heritage within migration contexts.

The shifting discourse regarding the preservation of Chinese heritage language features three distinct attitudinal stances in the context of a changing Chinese linguistic landscape. First, investing in the preservation of Chinese heritage language is strongly justified by the occupational opportunities and economic benefits it brings in the growing Chinese market. The material advantage of Chinese proficiency is rooted in China’s socio-economic significance on a global scale. The increasing commercialization of Chinese reflects the prevailing influence of neoliberalism in the language industry, which treats language as a commodity or resource in niche markets, prioritizing its economic importance (Liu and Gao, 2020; Curdt-Christiansen and Huang, 2021). This trend indicates that the commodification of the Chinese language is gaining popularity among grassroots diaspora communities, who consider heritage Chinese as a valuable asset. Second, preserving cultural heritage, ethnic identity, and fostering family bonds continue to be powerful drivers behind parental motivation for maintaining the Chinese language. However, the desire to preserve Chinese as a symbol of heritage, identity and intimacy is often exclusively entrusted to Mandarin Chinese, irrespective of participants’ diverse heritage varieties. As Mandarin is given privileged status as the recognized and proper carrier of cultural heritage, national identity and familial affection, other heritage varieties are marginalized, with the possibility of being overlooked or confined to the domain of familial interactions. Consequently, when Chinese languages are hierarchically ordered and restricted to specific social domains (Curran, 2021), the transmission of regional varieties, despite their connection to individuals’ ancestral heritage, becomes endangered or undermined. The notion of pride, commonly linked to Mandarin proficiency rather than non-Mandarin varieties, reflects the expansion of the commodifying ideology of the Chinese language into the realm of heritage and identity. Third, in addition to the limited opportunities of transmitting non-Mandarin Chinese varieties to the next generation, the long-term preservation of heritage Mandarin itself faces constant challenges. The difficulties in consistently committing to Mandarin proficiency stem from a linguistic battleground in which Mandarin, despite its strong economic potential, has lost ground within the diaspora community when competing with English—the globally dominant language atop linguistic hierarchies.

The research highlights that parental aspiration for maintaining Chinese as a heritage language is driven by both pragmatic considerations in response to the increasing prominence of the Chinese language, as well as emotional desires for cultural affiliation, identity recognition, and family unity. The study aligns with existing research on heritage language ideology, which emphasizes its three core values: the connection of heritage language to identity, family bonds, and the potential employability benefits (Curdt-Christiansen, 2014; Romanowski, 2022). What sets this study apart is its in-depth exploration of the interrelation, dynamics, and complexity of these three layers of ideology, exposing the commodification of Chinese heritage language bilingualism within grassroots family language policies. It sheds light on how a more instrumental conception of language takes precedence under the influence of socio-economic and socio-political factors within Chinese diaspora. Additionally, the study confirms the significance of hierarchical language order in family language planning, whereby societal languages are given priority at the expense of minority languages (Li G., 2006; Curdt-Christiansen, 2014). Furthermore, it illustrates power conflicts and ideological contradictions play out in the context of Chinese heritage language maintenance, as “tensions arise and hierarchical relations are formed” (Curdt-Christiansen and Huang, 2021, p. 70) during the implementation of family language policies. These power dynamics not only underscore the differentiation between Mandarin and non-Mandarin varieties but also reveal the nuanced stratifications present within different non-Mandarin languages.

This article extends the foundation laid by Heller and Duchêne (2012) in their conceptual framework, which revolves around the concepts of “pride” and “profit” within the sociocultural context of late capitalism and economic development. Our research has unveiled significant connections between language ideologies and practices and the evolving position of minority heritage languages in the discourse surrounding diaspora and transnationalism. As Heller and Duchêne (2012) previously associated the transition from “pride” to “profit” in minority language communities with the expansion of the new economy, our analysis draws a parallel between this ideological transformation and the growing prominence of China in the global market. The interplay between “pride” and “profit,” often observed in the context of heritage tourism catering to non-speakers of the local language (Jaffe, 2019; Tuktamyshova and Kirillova, 2023), takes on a distinctive dimension in this article. In the tourism sector, the national and regional identities that communities take pride in become commodified, commercialized, and packaged for sale to consumers who are usually outsiders to the local language and economy (Jaffe, 2019; Tuktamyshova and Kirillova, 2023). However, within the scope of this article, we highlight how the commodification of linguistic pride, as evidenced by the experiences of Chinese immigrant parents in Australia, is primarily driven by insiders’ pride in China’s rising global status and the increasing influence of Mandarin Chinese in the transnational sphere. This alternative perspective underscores the complex power dynamics in the realm of minority language preservation and the evolving roles of social actors in the era of late capitalism.

The current research into tensions in maintaining Chinese “heritage” languages and the “heritage” language underlines the previously identified social causes of minority language loss—“educational demands, public discourse and linguistic instrumentalism” (Curdt-Christiansen and Huang, 2021, p. 61). The research findings have important implications for language education, highlighting two key aspects. Firstly, the prevailing ideology that views languages primarily as commodities, valuing them based on their economic potential, does not support the sustainable development of any language or language variety (Liu and Gao, 2020), including Mandarin Chinese, despite its current high economic capital. The instrumental discourse surrounding the learning of Chinese heritage language undermines long-term prosperity in language education and maintenance, as its preference for profit-oriented motives fails to account for the complexities of our ever-changing world. Secondly, the pervasive ideology that prioritizes language as a means of profit further marginalizes many minority languages and poses a threat to their intergenerational transmission. While Mandarin Chinese, as a heritage language, may receive more policy support compared to other minority or community languages in Australia (Chen and Zhang, 2014), it still faces significant challenges in maintaining consistent usage when competing with the dominant presence of English. Consequently, the task of preserving other minority languages with less exchangeable value becomes even more arduous, making it increasingly difficult, if not impossible, to achieve the advocated goals of societal multilingualism and linguistic diversity.

In today’s globalized economy, it is impractical to completely disregard economic considerations in language planning. However, the maintenance of minority languages and the establishment of a sustainable multilingual society cannot be achieved unless language-in-education policies extend beyond national and economic interests to acknowledge the intrinsic values of languages (see also Liu and Gao, 2020). Therefore, in the context of the late capitalist era, it is more feasible to establish a mutually beneficial relationship between promoting language acquisition for national economic growth and personal advancement, while simultaneously preserving cultural heritage for the sake of national unity and intergenerational transmission. This approach can foster a positive synergy that combines economic objectives with the safeguarding of linguistic and cultural diversity.

Additionally, given the overrepresentation of mothers as study participants (23) compared to the limited number of fathers (4), it is reasonable to infer that the higher proportion of female participants may have influenced the ideological discourse surrounding notions of pride and profit in the context of the Chinese language. However, it is important to note that the pronounced gender imbalance among the participant parents does not significantly alter the overall landscape of parental ideological preferences. Throughout the interviews, it was observed that three out of the four interviewed fathers primarily emphasized the importance of learning Chinese in order to enhance their children’s competitive edge in the perceived growing Chinese market. Only one father, referred to as Father 3, did not clearly favor either identity preservation or profit generation when discussing the reasons of maintaining Chinese heritage language. These paternal attitudes toward Chinese language learning also align with the predominant ideological inclination among the study’s participants, which tends to prioritise profit-driven motivations over identity-related pride. Nevertheless, for a more comprehensive understanding of the ideological dynamics of heritage language bilingualism, future research should strive to include the perspectives of a greater number of fathers in the analysis of language ideologies and practices within diasporic communities.

## Data availability statement

The raw data supporting the conclusions of this article will be made available by the authors, without undue reservation.

## Ethics statement

The studies involving humans were approved by Macquarie University Human Research Ethics Committee. The studies were conducted in accordance with the local legislation and institutional requirements. The participants provided their written informed consent to participate in this study. Written informed consent was obtained from the individual(s) for the publication of any potentially identifiable images or data included in this article.

## Author contributions

YW: Validation, Writing – original draft. JL: Conceptualization, Writing – review & editing.

## Footnotes

^1^In this research, “intimate languages” are defined as those languages that are closely connected to people’s lives, serving them in both formal and informal communication contexts.

^2^Opportunity Class test is a test for admission into academically gifted and talented classes in Year 5 and 6 across NSW.

^3^The Selective High School Test is a placement test for admissions into highly competitive public schools for high-achieving and gifted students.

^4^HSC examination is the high school graduation examination in New South Wales.
