# Supplementary material for: Changing discourses of Chinese language maintenance in Australia: unpacking language ideologies of first-generation Chinese immigrant parents from People’s Republic of China
Source: Front Psychol. 2024 Jan 11;14:1259398. doi: 10.3389/fpsyg.2023.1259398 (PMC10808597; doi:10.3389/fpsyg.2023.1259398)
Supplement: Supplementary file 1 [file Data_Sheet_1.docx]

**Family questionnaire**

There are 3 sections in this questionnaire. Section 1 asks for some background information of parents. Section 2 mainly relates to your family’s language and migration experiences. Section 3 collects for information about the focal child.

**Section 1 Background information**

*About yourself*

1. What’s your name? ______________________
2. What’s your relationship with the child? □ father □ mother
3. What’s your age? □ 20-30 □ 30-40 □ 40-50 □ 50+
4. What is/are your first language/s?

□ Mandarin □ Cantonese □ English □ Other (please specify):______________

1. In which year did you arrive in Australia? ________________
2. What is your highest academic qualification?

□Primary □ High school/Secondary □ Bachelor □ Postgraduate

1. What was your occupation prior to migration?
2. What is your occupation now?

*About your spouse/partner*

1. What’s your name? ______________________
2. What is your age? □ 20-30 □ 30-40 □ 40-50 □ 50+
3. What is/are your first language/s?

□ Mandarin □ Cantonese □ English □ Other (please specify): _______________

1. In which year did you arrive in Australia? ________________
2. What is your highest academic qualification?

□Primary □ High school/Secondary □ Bachelor □ Postgraduate

1. What was your occupation prior to migration?
2. What is your occupation now?

**Section 2**  **Language and migration experiences**

*About your family*

1. What is your current citizenship status / your visa type? _______________________
2. How many children do you have? ______________________________
3. How many people in total live in your household? ___________________
4. What are the other adults living together except parents?

□Grandparents □ Other (please specify):__________________

1. What language/s do parents speak to each other at home? ______________________
2. What language/s do parents speak to child? _______________________
3. What language/s does your child speak to parents? ______________________
4. What languages do other adults at home speak to child? ____________________
5. What language/s does your child speak to other adults at home? ______________________
6. What language/s does your child speak to his/her siblings? _____________________
7. Which suburb are you living in now? ________________________
8. What were your reasons for migration? (you may choose more than 1 answer)

□a. Work/business

□b. Your (or your spouse/partner’s) education

□c. Your children’s education

□d. Family reunion

□e. Better living condition

□f. Political reasons

____________________________________________________________________

1. What is the best way to contact you?

Phone number: __________________________________

Email: _________________________________

**Section 3 General information about focal child**

1. What’s your child’s name?　＿＿＿＿＿＿＿＿＿＿＿＿
2. In which year was your child born? ______________________
3. In which year did your child arrive in Australia? _____________
4. In which type of school was your child prior to migration?

□ Chinese-medium public school

□ Bilingual Chinese-English school

□ Other (please specify):_______________

33. At what year/grade was your child prior to migration? ___________________

34. From which year/grade did your child start after he/she arrived in Australia? ___________

35. In which school is your child now? _______________________

36. Does your child go to a tutoring class / coaching college? □ Yes □ No

37. Does your child attend a Chinese Saturday school? □ Yes □ No
