# Supplementary material for: Changing discourses of Chinese language maintenance in Australia: unpacking language ideologies of first-generation Chinese immigrant parents from People’s Republic of China
Source: Front Psychol. 2024 Jan 11;14:1259398. doi: 10.3389/fpsyg.2023.1259398 (PMC10808597; doi:10.3389/fpsyg.2023.1259398)
Supplement: Supplementary file 2 [file Data_Sheet_2.docx]

**Interview Guide (for parents)**

**Phase 1** **Background information (before coming to Australia)**

1. Can you tell me about your English learning experiences before coming to Australia and how do you evaluate your English proficiency prior to migration?
2. Can you tell me about your child’s English learning and Chinese learning before coming to Australia and how do you evaluate your child’s English and Chinese proficiency prior to migration?
3. Can you tell me about your child’s education in China generally?
4. What kind of job did you do before moving to Australia? Can you tell me about your life back at home?
5. What made you come to Australia? What were your expectations of your child’s education abroad before coming to Australia?

## Phase 2 Education and adaptation in Australia

## *About parents*

1. Can you tell me about your experiences of English learning in Australia?
2. What kind of job do you do now?
3. Can you tell me about your social activities and about your social circles?
4. Did you see some changes in your life after moving to Australia in terms of your job opportunities, living standard, education opportunities and social activities?
5. What are your happiest experiences (e.g. in education and life) after moving to Australia?
6. What are some of the problems and difficulties you have experienced in Australia?
7. How much are Chinese values a part of your life (e.g. Chinese language, Chinese books and programs, Chinese food and festivals)?
8. How much are mainstream Australian values a part of your life (e.g. English speaking, English reading and programs, Australian sports, Australian food and festivals)?
9. What do you think of your identity (more Chinese or Australian)?
10. Do you have education goals, career goals and/or other goals for yourself? How do you work towards them?

*About child’s school education*

1. Can you tell me about your child’s school? Why do you choose this particular school for your child?
2. Did your child receive any language support from school (e.g. ESL program)? What kind of support did he/she receive and what do you feel about it?
3. Can you tell me about your child’s subject learning in Australian schools? Can you tell me about your child’s school performance compared with that back home?
4. Does your child go to a tutoring class/coaching college, or have a private tutor or any other support outside school? What’s your reason for sending your child to these classes?
5. How satisfied are you with your child’s education compared with that back home? / What educational aspects are you satisfied and dissatisfied with?
6. Do you see some changes of your expectations towards your child’s education? If so, what kind of changes do you experience and why? What kind of education generally do you want for your child and how do you work towards that?

*About child’s heritage language practice and bilingual abilities*

1. Can you tell me about your child’s heritage language proficiency and English language proficiency before and after moving to Australia?
2. How do you view the status of your child’s heritage language ability and English language ability?
3. What languages do parents use to the child? What languages does the child use to parents and to siblings? Are there some changes in language use at home and what are those changes? What do your think are the main factors leading to language changes?
4. Do you have a family language policy? What’s your family language policy and is it easy or difficult to implement your family language policy?
5. Did you try/Are you trying to maintain your child’s heritage language? What’s the heritage language education goal for your child?
6. Is English language learning an issue in your family? How do you deal with this issue?
7. What is your expectation of your child’s language abilities (bilingual abilities)?

*About child’s social, emotional, and cultural adaptation*

1. In which suburb and/or community do you live? Why did you choose to live there?
2. Can you tell me about your child’s socializing activities / after-school activities?
3. How important do you view Chinese friends and mainstream Australian friends respectively to your child? What is your child’s social circle?
4. What does your child feel about Australian schools, his/her friends here and his/her host country?
5. How important is Chinese culture and mainstream Australian culture respectively to your child? What did you do for that (e.g. festival celebration, book reading, food choices) and what do you think is your child’s cultural identity?
6. Can you tell me about the relationship between you and your child? Have you experienced any changes of your parent-child relationship between pre- and post-migration? If so, what do you think are the main reasons of these changes?
7. What is your expectation of your child’s identity formation?

**Interview Guide (for child)**

**Phase 1** **Background information (before coming to Australia)**

1. Do you still remember what grade you did finish in China?
2. What subjects did you like when you were in China? Can you tell me about your school life back in China?
3. Can you tell me about your English learning in China?
4. What did you usually do outside school? What kind of after-school activities did you enjoy?
5. Can you tell me something about your friends and family members back at home?
6. Who looked after your when you were in China?
7. Did you want to come to Australia? What was your expectations of this new country?

## Phase 2 Life in Australia

## *About social life*

1. Did you like Australia when you first came here and what do you feel now?
2. Can you tell me about your best/worst experience or some funny / embarrassing experiences since coming to Australia?
3. How do you spend your time outside school? What kind of activities do you like most?
4. Do you have many friends here? What languages do you speak to your friends?
5. Do you miss your friends and family members back in China? How do you keep contact with them?
6. Who is looking after you in Australia? What languages do you speak to them?

*About languages*

1. Can you tell me about your English learning in Australia? What kind of language support did you get from and outside your school?
2. Do you still learn your first language? Do you want to improve your first language?
3. What languages do your parents speak to you? What languages do you use to parents and to siblings? What languages do you prefer to use?
4. Do you read English and/or Chinese books? Do you watch English and/or Chinese movies or programs?

*About subject learning*

1. How do you feel about Australian schools? What do you like about them? What do you dislike about them?
2. Do you see some differences between your Australian school and your school back in China?
3. What subjects do you like in Australian schools? Are there some subjects which you find difficult?
4. Do you need to go to a tutoring class or a coaching college? What do you learn there?
5. How much homework do you need to do?

*About future plan*

1. What do you want to do in the future? / What kind of school do you want to go? How do you work towards your dream?
